# Human glioma stem-like cells induce malignant transformation of bone marrow mesenchymal stem cells by activating TERT expression

**DOI:** 10.18632/oncotarget.22301

**Published:** 2017-11-06

**Authors:** Yaodong Zhao, Jinsheng Chen, Xingliang Dai, Honghua Cai, Xiaoyan Ji, Yujing Sheng, Hairui Liu, Lin Yang, Yanming Chen, Dengguo Xi, Minfeng Sheng, Yanping Xue, Jia Shi, Jiachi Liu, Xiaonan Li, Jun Dong

**Affiliations:** ^1^ Brain Tumor Research Laboratory, 2nd Affiliated Hospital of Soochow University, Suzhou, Jiangsu Province 215004, China; ^2^ Department of Neurosurgery, Shanghai General Hospital, Shanghai Jiaotong University, School of Medicine, Shanghai 200080, China; ^3^ Department of Neurosurgery, People’s Hospital of Susong, Susong, Anhui Province 246500, China; ^4^ Department of Neurosurgery, 2nd Affiliated Hospital of Soochow University, Suzhou, Jiangsu Province 215004, China; ^5^ Laboratory of Molecular Neuro-oncology, Texas Children’s Cancer Center, Baylor College of Medicine, Houston, TX 77030, USA

**Keywords:** glioma stem-like cells, bone marrow mesenchymal stem cells, TERT, malignant transformation

## Abstract

We investigated whether glioma stem-like cells (GSCs) malignantly transformed bone marrow mesenchymal stem cells (tBMSCs) in the tumor microenvironment. Transplantation of enhanced green fluorescence protein (EGFP)-labeled BMSCs into irradiated athymic nude mice was followed by intracranial injection of red fluorescent protein-expressing glioma stem-like cells (SU3-RFP-GSCs). Singly cloned EGFP-BMSCs, harvested from the intracranial tumors showed TERT overexpression, high proliferation, colony formation and invasiveness in Transwell matrigel assays. Transfection of normal BMSCs with TERT (TERT-BMSCs) enhanced proliferation, colony formation and invasiveness, though these characteristics remained lower than in tBMSCs. The tBMSCs and TERT-BMSCs showed high surface expression of CD44, CD105, CD29 and CD90 and an absence of CD31, CD34, CD45, and CD11b, as in normal BMSCs. Alizarin red S and oil red O staining confirmed tBMSCs and TERT-BMSCs transdifferentiated into osteocytes and adipocytes, respectively. When normal BMSCs were indirectly co-cultured in medium from SU3-RFP-GSCs, they exhibited increased growth and proliferation, suggesting paracrine factors from GSCs induced their malignant transformation. Tumorigenicity assays in athymic nude mice showed that transplanted tBMSCs and TERT-BMSCs generated 100% and 20% subcutaneous tumors, respectively, while normal BMSCs generated no tumors. GSCs thus induce malignant transformation of BMSCs by activating TERT expression in BMSCs.

## INTRODUCTION

Gliomas are the most malignant primary intracranial tumor [1]. Recent studies have shown that the interaction between glioma stem-like cells (GSCs) and tumor stromal cells in the tumor microenvironment is critical for the development and progression of gliomas [2, 3]. Bone marrow mesenchymal stem cells (BMSCs) are recruited to the tumor microenvironment and act as tumor stromal cells [4, 5]. BMSCs are a class of adult stem cells that directly migrate to sites of injury, inflammation, and tumors to promote tissue repair and regulate immune regulation. Therefore, they are extensively used as cell vectors in treatment of multiple diseases including tumors. A combination of interferon β expressing BMSC (IFN β-BMSCs) transplantation and temozolomide showed more effective inhibition of GL26 mouse glioma cell proliferation than temozolomide treatment alone [6]. However, the therapeutic use of the BMSCs is minimized because some studies have reported that normal BMSCs generate tumors and promote metastasis [7, 8]. On the contrary, other studies have shown that normal BMSCs inhibit tumor cell proliferation and angiogenesis [9, 10]. Kanehira et al performed *in vivo* and *in vitro* studies and reported no evidence for BMSC transformation [11]. But, Liu et al. reported malignant transformation of BMSCs by indirectly co-culturing them with C6 rat glioma cells [12]. In our previous studies, GSCs induced malignant transformation of tumor stromal cells such as oligodendrocytes [13], macrophages [14], and fibroblasts [15] in the tumor microenvironment (TME). In this study, we investigated if GSCs induced malignant transformation of BMSCs and the underlying molecular mechanisms involved in this process.

## RESULTS

### RFP^+^ GSCs and GFP+ BMSCs co-operate in tumor tissue remodeling

**Figure 1:**
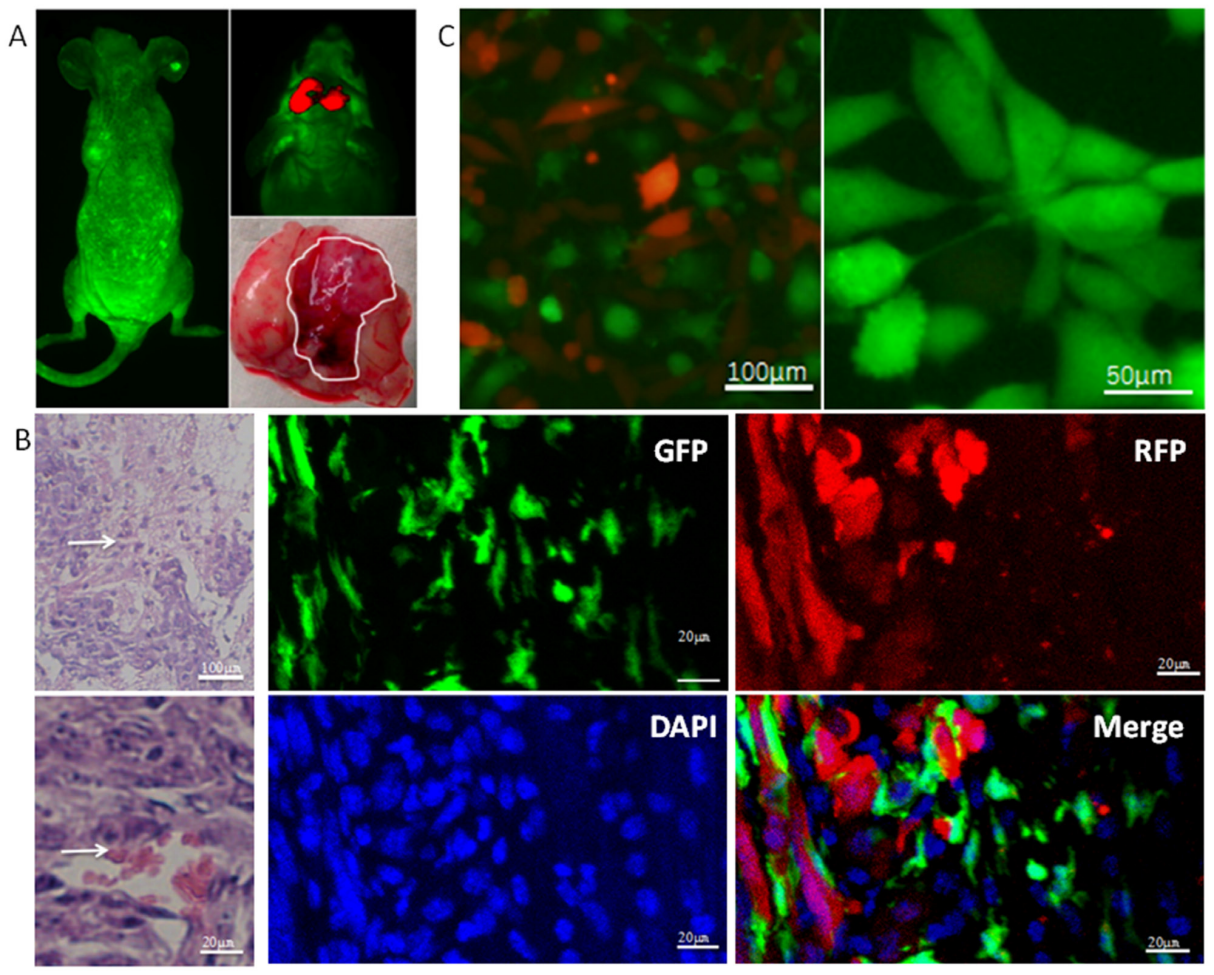
Characterization of SU3-RFP GSC and EGFP-BMSC interactions in intracranial xenograft tumors **(A)** Top left shows live fluorescence image of a irradiated mouse transplanted with bone marrow derived GFP^+^ cells. The light green fluorescence is seen all over whole body. Top right shows SU3-RFP derived intracranial tumor (red). Bottom right shows the whole brain with the white trace showing tumor derived from SU3-RFP cells. **(B)** Top left shows H&E stained SU3-RFP derived intracranial tumor sections with densely arranged tumor cells interspersed with blood vessels (white arrow). Bottom left image shows red blood cells in the vessel lumen (white arrow) of SU3-RFP derived intracranial tumor sections. Right images show laser scanning confocal microscopic images of the SU3-RFP derived intracranial tumor sections showing exogenous bone marrow cells (green) interacting with SU3-RFP tumor cells (red) in the tumor parenchyma (bar: 20μm). **(C)** Fluorescence images (left) of primary culture of SU3-RFP xenograft tumor tissue derived cells showing both SU3-RFP cells (red) and bone marrow-derived GFP^+^ cells (green; bar: 100μm). Fluorescence images (Right) showing highly proliferating GFP^+^ cells with high proliferative ability that were derived from a single cell by micro-pipetting techniques (bar: 50μm).

We established an *in situ* xenograft GSC-tumor model by intravenously injecting EGFP-BMSCs and intracranially injecting SU3-RFP human GSC cells in irradiated Balb/c nude mice (Figure 1A). Live fluorescence imaging showed non-homogenous light green fluorescence (EGFP-BMSCs) throughout the mice at 4 weeks and red fluorescent (SU3-RFP-GSCs) intracranial xenograft tumors (Figure 1A). H&E staining of xenograft tumor sections showed high cellular density, cells with irregular hyperchromatic nuclei, rich blood supply, and necrotic hemorrhagic foci characteristic of tumors (Figure 1B). Immunofluorescence images showed that the tumor cells expressing RFP and EGFP- BMSCs were in close proximity in the tumor tissue (Figure 1B). This suggested that the implanted exogenous bone marrow cells were involved in tumor tissue remodeling in association with the GSCs.

### Characterization of GFP^+^ BMSCs derived from xenograft intracranial tumors

**Figure 2:**
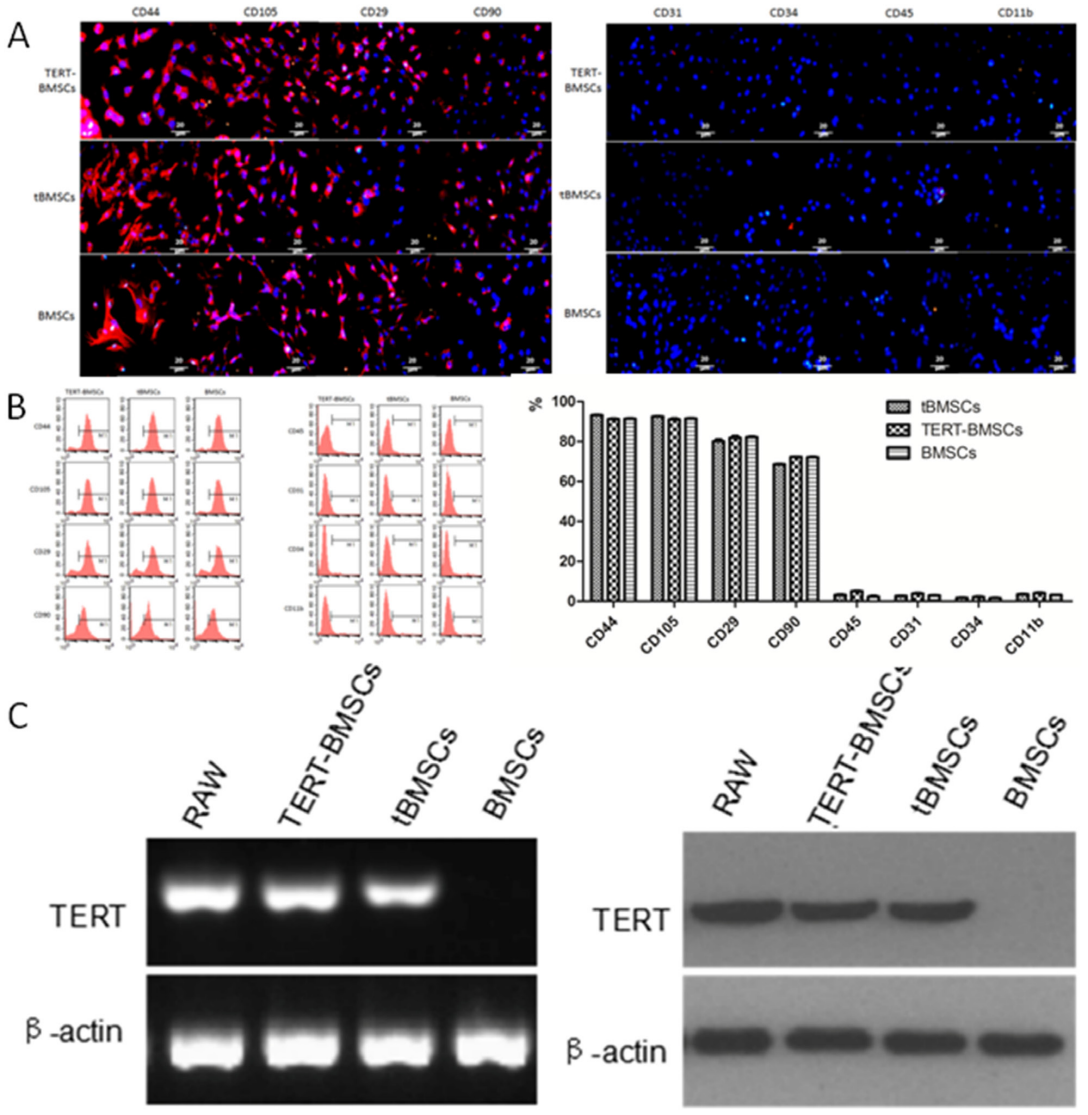
Characterization of t-BMSCs, TERT-BMSCs and normal BMSCs **(A)** Representative images show immunofluorescence staining of CD44, CD105, CD29, CD90, CD31, CD34, CD45, and CD11b cell surface markers in TERT-BMSCs, tBMSCs, and BMSCs. As shown, all 3 BMSCs express high levels of CD44, CD105, CD29 and CD90, but do not express CD31, CD34, CD45, and CD11b (bar: 20μm). **(B)** Representative FACS plots show cell surface staining of CD44, CD105, CD29, CD90, CD31, CD34, CD45, and CD11b in TERT-BMSCs, tBMSCs, and BMSCs. As shown, all 3 BMSCs TERT showed high expression of CD44, CD105, CD29, and CD90, but did not express CD31, CD34, CD45, or CD11b. Therefore, tBMSCs and TERT-BMSCs retain differentiation markers of BMSCs. **(C)** RT-PCR (left) and western blot (right) analysis show TERT mRNA and protein expression in TERT-BMSCs, tBMSCs, and BMSCs. RAW264.7 cells were used as positive control for TERT. β-actin was used as internal control. TERT-BMSCs and tBMSCs show high expression of TERT than normal BMSCs.

Primary culture of single cell suspension from the xenograft tumor tissues showed both red and green fluorescent cells (Figure 1C-left). The GFP^+^ cells derived from the tumors showed clonal properties and could be subcultured continuously (Figure 1C-right). Next, we performed immunofluorescence staining of various cell surface markers, including BMSC-specific markers to characterize the highly proliferative single cell derived GFP^+^ bone marrow cells. We observed that the highly proliferative GFP^+^ cells cloned from xenograft tumors were similar to TERT-BMSCs and normal BMSCs and showed high CD44, CD105, CD29 and CD90 expression and very low CD31, CD34, CD45, and CD11b expression (Figure 2A). Since this expression profile was similar to BMSCs, we designated the highly proliferating GFP^+^ cells from xenograft tumors as transformed BMSCs (tBMSCs). Flow cytometry analysis of tBMSCs, TERT-BMSCs, and BMSCs also demonstrated high CD44, CD105, CD29 and CD90 expression and very low CD31, CD34, CD45, and CD11b expression (Figure 2B). This suggested that the transplanted normal BMSCs underwent malignant transformation *in vivo* or when transfected with TERT gene *in vitro*. This resulted in enhanced proliferation capacity of these transformed BMSCs while maintaining the characteristics of normal BMSCs. CD29 and CD90 staining in tBMSCs was non-homogenous reflecting heterogeneity in the transformed tBMSCs.

### Transformed BMSCs demonstrate high TERT expression

Next, we analyzed TERT mRNA and protein expression in tBMSCs, TERT-BMSCs and normal BMSCs by RT-PCR and western blotting, respectively. We observed high TERT mRNA and protein levels in BMSCs stably transfected with TERT gene (TERT-BMSCs) and tBMSCs than in normal BMSCs (Figure 2C). The mouse RAW264.7 macrophage cell line with high TERT expression was used as a positive control (Figure 2C).

### Transdifferentiation of tBMSCs, TERT-BMSCs and normal BMSCs into osteocytes and adipocytes

**Figure 3:**
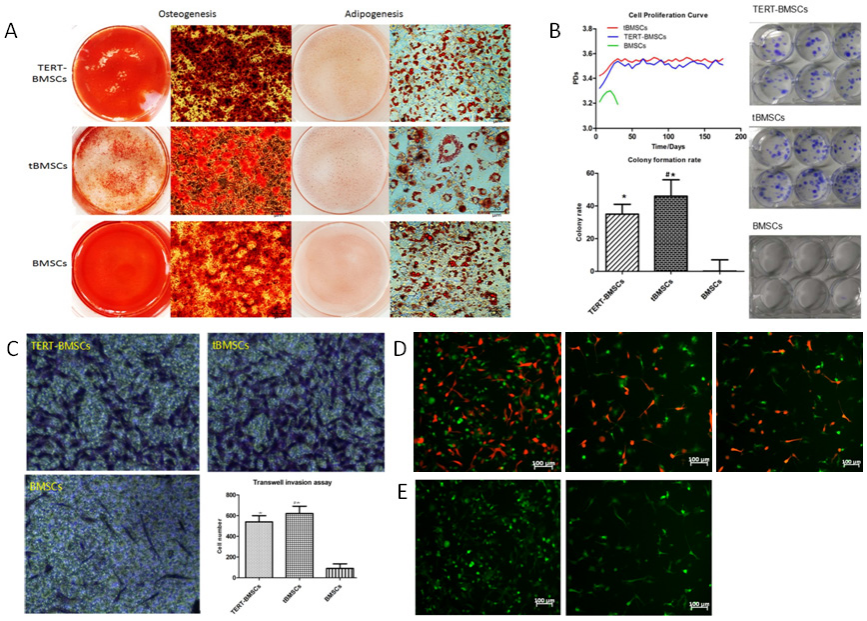
*In vitro* characterization of TERT-BMSCs, t-BMSCs and normal BMSCs *in vitro* **(A)** Representative images of Alizarin Red S and Oil Red O staining of TERT-BMSCs, tBMSCs, and BMSCs differentiated into osteocytes (left) and adipocytes (right) respectively. (bar: 20μm). **(B)** Growth curves of TERT-BMSCs, tBMSCs and normal BMSCs show that both TERT- and tBMSCs show increased proliferation demonstrated by population doubling (PD). However, PD values for normal BMSCs gradually decreases after 5 generations. (Right) Colony formation assay shows that TERT-BMSCs form lower number of colonies than tBMSCs, but higher than normal BMSCs. Note: ^*^ denotes p<0.05 when TERT-BMSCs/tBMSCs are compared with BMSCs (top left); ^#^ denotes p<0.05 when TERT-BMSCs and tBMSCs are compared (lower left). **(C)** Representative images showing results of the Transwell invasion assay. As shown by crystal violet staining of fixed BMSCs that invade the matrigel in all 3 groups, TERT-BMSCs show less invasiveness than tBMSCs (^*^ p<0.05), but higher invasiveness than normal BMSCs (^#^ p<0.05). **(D)** Representative fluorescent images showing results of the *in vitro* co-culture of SU3-RFP cells with BMSC-derived cells at a ratio of 1:5. At two weeks, both SU3-RFP and tBMSC cell numbers increase significantly (left). Both SU3-RFP and TERT-BMSCs show mild increase in cell density when co-cultured (middle). SU3-RFP cells and BMSCs co-cultured for two weeks show low cell density, though SU3-RFP keep actively growing (right). **(E)** Representative fluorescent images showing results of the *in vitro* indirect co-culture of BMSC-derived cells with (left) or without (right) SU3-RFP culture medium (bar 100μm). As shown, BMSCs proliferate faster with supernatant of SU3-RFP culture medium (left) as indicated by higher numbers, thereby suggesting paracrine mechanism.

Next, we analyzed the transdifferentiation properties of tBMSCs, TERT-BMSCs and normal BMSCs. As shown in Figure 3A, the trans-differentiated TERT-BMSCs, tBMSCs, and BMSCs showed positive staining for Alizarin Red S (osteocyte-positive) and Oil Red O (adipocyte-positive). However, tBMSCs demonstrated lower density and quantity of red calcium nodules than TERT-BMSCs and BMSCs. Moreover, the multilineage differentiation capacity negatively correlated with the degree of malignancy of the BMSC derived cells. The tBMSCs showed least multilineage differentiation and proliferated more rapidly and became non-adherant during transdifferentiation into either osteocyte or adipocyte lineages, whereas the normal BMSCs were adherent throughout the transdifferentiation process.

### Biological features of TERT-BMSCs, tBMSCs, and BMSCs

Next, we analyzed the differences in biological characteristics of TERT-BMSCs, tBMSCs, and BMSCs by growth curves, colony formation, and transwell invasion assays. TERT-BMSCs showed lower proliferation, colony formation rate, and invasiveness than tBMSCs (p<0.05). Moreover, both TERT-BMSCs and tBMSCs showed higher proliferation, colony formation and invasiveness than normal BMSCs (p<0.05; Figure 3B-3C).

Next, we co-cultured SU3-RFP GSCs with TERT-BMSCs, tBMSCs and BMSCs in a 1:5 ratio for two weeks. We observed significant interaction between SU3-RFP-GSCs and tBMSCs with enhanced proliferation and growth of both cells (Figure 3D, left). The co-culture of SU3-RFP cells with TERT-BMSCs demonstrated mild increase in cell number of both cell types (Figure 3D-middle). When SU3-RFP cells were co-cultivated with BMSCs, although SU3-RFP cells showed active growth, the normal BMSCs did not proliferate much and their cell number was lower than both tBMSCs and TERT-BMSCs (Figure 3D-right). Moreover, when normal BMSCs were grown in the supernatant of SU3-RFP cultures, they demonstrated increased proliferation as demonstrated by their higher cell numbers, thereby implying the role of paracrine factors (Figure 3E).

### Transplanted t-BMSCs and TERT-BMSCs generate subcutaneous tumors in nude mice

**Figure 4:**
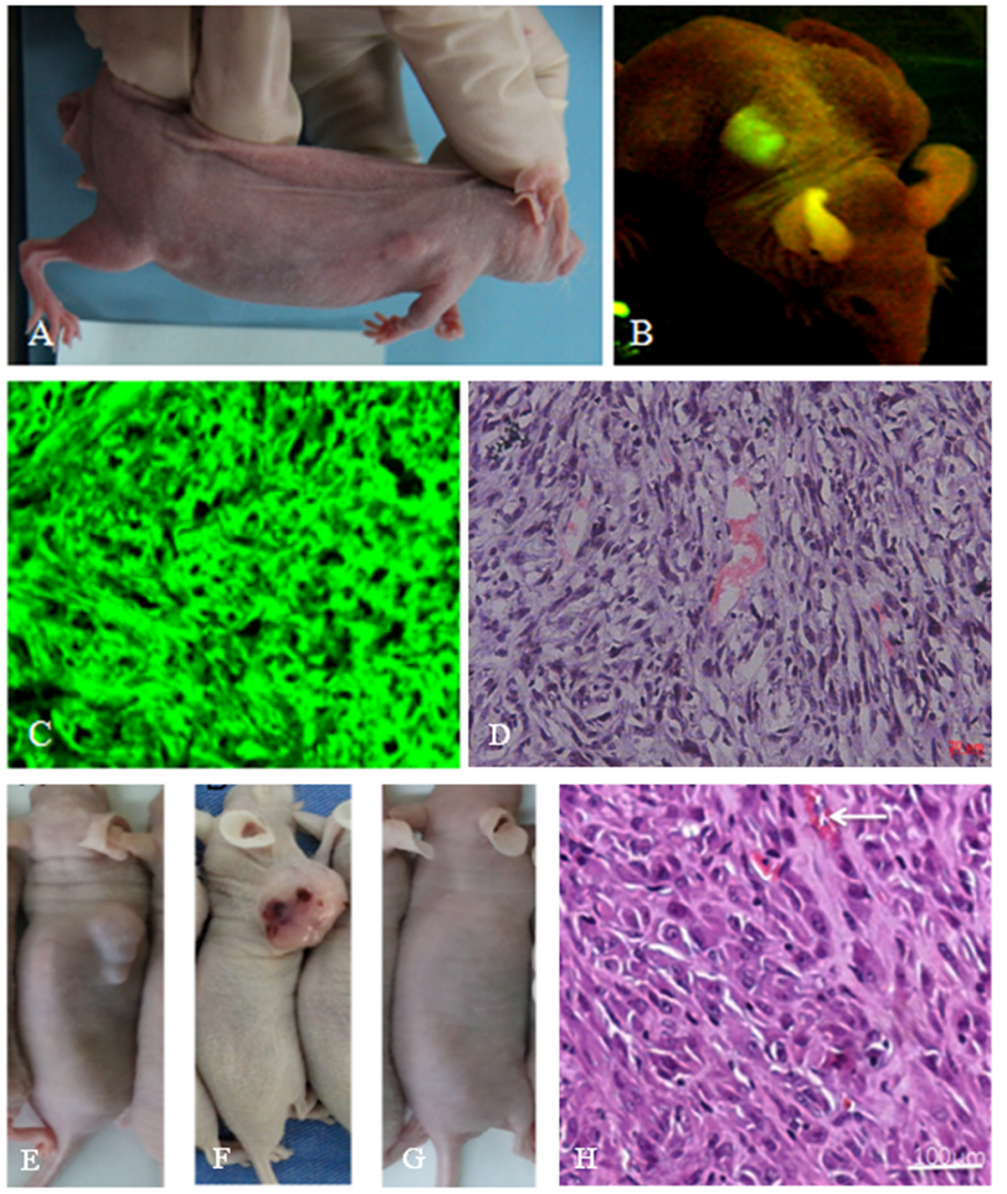
*In vivo* tumorigenicity assay of tBMSCs, TERT-BMSCs and normal BMSCs **(A)** Representative image of a nude mouse transplanted with tBMSCs that produced subcutaneous tumors. All 10 nude mice transplanted with tBMSCs produce subcutaneous tumors in four weeks. **(B)** Live fluorescence imaging showing green fluorescence from the subcutaneous tumors derived from the tBMSCs. **(C)** Fluorescence imaging of a tumor section derived from the tBMSC transplanted nude mice. **(D)** H&E staining of tBMSC-derived tumor section shows disordered cell arrangement; tumor cells have big heterotypic nuclei with deep stained chromatin; cell proliferation is visible with rich blood vessels. **(E-G)** Gross morphology of tumors after subcutaneous implantation of TERT-BMSCs, tBMSCs, and BMSCs in nude mice. Note: subcutaneous implantation of BMSCs did not produce any visible tumor (G). **(H)** H&E stained TERT-BMSC-derived tumor tissue section showed tumor cells with big obvious heterotypic nuclei and deep stained chromatin.

We conducted *in vivo* tumorigenicity experiments in nude mice to evaluate if tBMSCs and TERT-BMSCs generated tumors and their efficiency. After 4 weeks of transplantation, tBMSCs generated visible subcutaneous tumors in all 10 mice (100%). In case of TERT-BMSCs, only 2 out of 10 mice developed tumors (diameter larger than 1 cm) in 4 months after transplantation (20%). Transplanted BMSCs did not generate any tumors in 4 months. H&E staining of xenograft tumor tissue sections generated from TERT-BMSCs and tBMSCs showed dense tumor cells with large irregular hyperchromatic nuclei that were disorganized (Figure 4). Besides, the tBMSC-derived tumors also showed angiogenesis as demonstrated by blood vessels and necrotic hemorrhagic lesions (Figure 4).

## DISCUSSION

The two different types of stem cells in the bone marrow are the hematopoietic stem cells (HSCs) that generate different blood cell types and bone marrow mesenchymal stem cells (BMSCs). The BMSCs play a critical role in immune regulation and tissue regeneration. Hence, they have been extensively used in preclinical studies involving regenerative medicine, inflammatory diseases, autoimmune diseases, and target therapy of graft-versus-host diseases and tumors [16, 17]. However, it is controversial if BMSCs exhibit anti-tumor or tumor functions. Therefore, to explore the exact role of BMSCs in GSC-initiated tumorigenesis and development, we first implanted GFP-BMSCs from GFP+ nude mice into irradiated nude mice and then intracranially injected RFP-labeled human glioma stem-like cell line (SU3-RFP) to establish a murine brain tumor model.

We demonstrated highly proliferative and clonal GFP^+^ stromal cells from xenograft tumors. BMSCs typically express CD29, SH2 (CD105), SH3 and SH4 (CD73), CD44, CD90, and CD166 cell surface markers, but lack common hematopoietic and endothelial markers such as CD11b, CD14, CD31, CD34, and CD45. We demonstrated that the highly proliferative and clonal GFP^+^ cells that were derived from exogenously implanted GFP^+^ bone marrow cells were derived from BMSCs based on cell surface marker analysis with an ability to differentiate along the osteocyte and adipocyte lineages. They also showed increased *in vitro* proliferation, colony formation and invasion properties and generated *in vivo* subcutaneous tumors in mice. Therefore, the BMSCs that underwent malignant transformation by SU3-RFP-GSCs were termed as transformed bone mesenchymal stem cells (tBMSCs).

The molecular mechanism underlying the malignant transformation of BMSCs during recruitment and GSCs-initiated tissue remodeling of xenograft tumors is unknown. One possible candidate is the TERT protein, which is subunit of telomerase and is highly expressed in tumor cells [18]. TERT promotes migration [19], and invasion of tumor cells [20]. Moreover, TERT is utilized for molecular sub-classification of gliomas [21, 22] and is a prognostic and predictive molecular marker of glioma [18]. Our results showed that TERT was highly expressed in tBMSCs, whereas normal BMSCs did not express TERT. Stable transfection of wild-type TERT into normal BMSCs (TERT-BMSCs) resulted in its over-expression and low transformation of BMSCs.

Recently, recurrent mutations in the promoter region of TERT were detected in 70% of malignant melanomas [23, 24]. Importantly, these promoter mutations were associated with TERT overexpression and subsequent tumorigenesis [24, 25]. Besides, TERT promoter mutations were associated with poor clinical outcomes in glioblastoma patients and in lower-grade gliomas [26, 27]. TERT promoter mutations have been used for classification and prognostic prediction in lower grade gliomas [28]. Therefore, overexpression of TERT is a biomarker for glioma recurrence, prognosis and progression. Our studies showed that indirect co-culture of BMSCs with supernatant of GSC culture promoted proliferation of GFP^+^ BMSCs. This suggested that paracrine mechanism may be involved in malignant transformation of BMSCs by GSCs.

Several studies have reported potential application of BMSCs in anti-tumor therapy. A combination of interferon β expressing BMSCs and temozolomide treatment effectively inhibited proliferation of GL26 mouse glioma cells [6]. In this case, BMSCs served as carriers for IFN-β to target tumor cells. Moreover, IFN-β suppresses tumors by inhibiting TERT transcription [29]. Thus, although GSCs promote TERT expression in BMSCs recruited to the tumors, large amount of IFN-β produced by IFN β-BMSCs inhibits TERT transcription, thereby preventing transformation of the IFN β-BMSCs. In another study, human mesenchymal stem cells exerted anti-tumorigenic effects in a model of Kaposi’s sarcoma by inhibiting Akt activation in tumor cells, but had no effect when incubated with prostate tumor cell line PC-3 or breast cancer cell line MCF-7 suggesting cell specific inhibition of the Akt protein kinase [9]. In addition, MSCs demonstrated a concentration- dependent inhibition of angiogenesis in a model of subcutaneous melanoma [10]. On the contrary, another study showed that MSCs promoted angiogenesis [10].

Our data suggests that GSCs induce transformation of BMSCs *in vivo*. The tBMSCs promote GSC proliferation more effectively than TERT-BMSCs and normal BMSCs suggesting specific interactions during tumor development. Since BMSCs have limited proliferation potential, they have not been widely applied in clinical and pre-clinical studies. Therefore, BMSCs have been immortalized with TERT transfection or other genetic elements. But our results demonstrate that TERT overexpression promotes transformation in 20% of the cases. It also implicates TERT as a factor for promoting stromal cells transformation during GSCs-initiated tumorigenesis such as fibroblasts, immune cells, vessels and other cells in the tumor microenvironment. This aspect needs to be investigated in detail. In conclusion, our study demonstrates that GSCs transform BMSCs in a tumor microenvironment. Also, TERT is one of the factors that are essential for malignant transformation of BMSCs by GSCs.

## MATERIALS AND METHODS

### Ethics statement

Investigations were conducted in accordance with the ethical standards of Declaration of Helsinki and approved by the Medical Review Board of Soochow University Medical School. All procedures were conducted in accordance with Chinese laws governing animal care.

### Cell lines, lentiviral vectors and nude mice

The TERT gene clone (Gene ID: NM_198253.2), and TERT over-expression lentiviral vector (pCDH-CMV-MCS-EF1-TERT-puro) were obtained from Shanghai Innovation Biotechnology Co. Ltd., China. The RFP gene in lentiviral vectors (Genechem Chemical Technology Co., Ltd., Shanghai, China) was transfected into human GSC cell line SU3 cells [15] and high and stably expressing SU3-RFP were obtained as previously reported [14]. SU3-RFP cells were cultured in DMEM/F12 (Gibco-Invitrogen) containing 20 ng/ml recombinant human basic fibroblast growth factor (PeproTech, Princeton, NJ, USA) and recombinant human epidermal growth factor (PeproTech). EGFP-Balb/c nude mice expressing EGFP (6-8 weeks old, body weight 23-26g) were established as previous reports [30]. Both EGFP-Balb/c and Balb/c nude mice (6-8 weeks old, body weight 23-26g) were bred and maintained in a specific pathogen-free (SPF) animal care facility. RAW 264.7 macrophage cell line was obtained from ATCC (Manassas, USA) and cultured in DMEM medium supplemented with 10% fetal calf serum.

### *In vitro* BMSC culturing protocol

BMSCs were cultured with whole bone marrow adherence culture method as described previously [31]. Briefly, EGFP-Balb/c nude mice were sacrificed by cervical dislocation after general anesthesia. The femur and tibia were immersed in 75% ethanol for 5-10min and then separated under sterile conditions by peeling off the attached skin, fascia, and muscle. After washing with PBS buffer containing 1% penicillin –streptomycin mixture, the metaphyses of femur and tibia were cut open. The bone cavity was repeatedly flushed with a syringe filled with MUCMX-90011 complete culture medium until the bone became transparent. Then, the mixture of bone marrow cells were cultured in a plastic flask at 37°C in a 5% CO_2_ incubator with the appropriate amount of MUCMX-90011 complete culture medium. After 72h, half of the culture medium was removed, and an equal volume of new complete culture medium was added. Non-adherent cells were discarded. The culture medium was replaced every 3 days. Cells were passaged when cell density reached 90% confluence and prepared for subsequent experiments.

### Transplantation of EGFP- BMSCs into Balb/c nude mice

The Balb/c nude mice were irradiated with an X-ray dose of 6Gy as previously described [32]. Twenty-four hours after irradiation, the femur and tibia of non-irradiated EGFP-Balb/c nude mice were separated under sterile conditions, and bone marrow cells were flushed out with PBS buffer, filtered and centrifuged followed by red blood cell lysis. Then, the cells were resuspended in serum-free DMEM/F12 culture medium. Bone marrow cells (1×10^7^ cells/mouse) were transplanted via tail vein injection. After transplantation, animals were housed in specific-pathogen-free animal care facility.

### *In situ* intracranial tumor model

Bone marrow destruction and reconstruction was performed as previously described [33]. Briefly, mice were anesthetized with 10% chloral hydrate (4-5ml/kg body weight). A 0.5 cm long longitudinal incision was made at 1.0 mm anterior to bregma and 2.5 mm away from right of the midline and the skull was drilled with a microscopic drill to expose the dura. Then, 1×10^5^ SU3-RFP cells in 20μl of serum-free culture medium were gradually injected into the right caudate nucleus of mouse brain for over 10 minutes with a micro-injector, which was vertically inserted 4.0 mm deep into the brain through the bur hole and then the needle tip was retreated by 1.0mm. Tumor tissue and host brain biopsies were obtained when mice exhibited any signs. Serial frozen sections were prepared after tissues were cryopreserved in 20% sucrose and 30% sucrose solutions. Sections were stained with hematoxylin and eosin (H&E) and observed under a microscope. In addition, other frozen sections were stained with DAPI and were observed and photographed under a laser scanning confocal fluorescence microscope.

### Single cell cloning of primary EGFP-transformed BMSCs from intracranial tumors

Tumor xenografts were washed with PBS buffer containing 1% penicillin and streptomycin. The tissues were minced and digested with 0.25% trypsin. The cells were obtained after filtering through a 40-μm mesh filter, and centrifugation followed by resuspension in DMEM/HG culture medium containing 10% fetal bovine serum (FBS). EGFP^+^ cells were separated with flow cytometry, and single EGFP^+^ cells with high proliferation ability were mono-cloned by micropipetting techniques and transferred into a 96-well plate for continuous culture. The cloned EGFP^+^ cells with high proliferation capacity were designated as transformed bone mesenchymal stem cells (tBMSCs).

### Immunofluorescence staining for cell surface marker analysis

BMSCs and tBMSCs that were growing logarithmically were adjusted to a density of 5×10^4^ cells/ml and cultured on cover slips overnight. Then, cells were fixed in 4% paraformaldehyde for 20min, permeabilized in 0.5% Triton X-100 at room temperature for 20min and incubated with 0.3% H_2_O_2_ at 37°C for 30min. Then, the cells were blocked with 5% serum at 37°C for 20min followed by incubation at 4°C overnight with the following primary antibodies from Abcam, UK: (1) CD105, 1:200, clone number 8A1; (2) CD90, 1:200, clone number IBL-6/23; (3) CD44, 1:150, clone number T2-F4; (4) CD29, 1:150, clone number KM16; (5) CD45, 1:200, clone number IBL-3/16; (6) CD34, 1:200, clone number ICO-115; (7) CD-11b, 1:200, clone number EPR1344, and (8) CD31, 1:200, clone number: JC/70A. Next day, after washings with immune staining solution (P0106C, Beyotime, Shanghai, China), the cells were incubated with secondary antibodies of Cy3-labeled Goat Anti-Rabbit/Rat IgG (H+L) (1:500, Beyotime, Shanghai, China) at 37°C for 1h followed by staining with DAPI Staining Solution DAPI (C1005, Beyotime, Shanghai, China) for 3-5min. Finally, they were mounted in fluorescence quenching solution and observed under a fluorescence microscope (MF53, Mshot, Guangzhou, China).

### Construction of pCDH-CMV-MCS-EF1-TERT-puro vector

The human telomerase reverse transcriptase (TERT) primers were designed based on its mRNA sequence available from the GenBank (Gene ID: NM_198253.2) along with XbaI and EcoRI restriction sites at both 5’ and 3’ ends. The target fragment was PCR amplified and purified from agarose gel after electrophoresis. The primers used for PCR amplification were 5’- CTA GTC TAG ACT AGA TGC CGC GCG CTC CCC GCT GCC GAG -3’, and 5’- CCG GAA TTC CGG TCA GTC CAG GAT GGT CTT GAA GTC T-3’ for TERT, where the size of the fragment PCR amplified was 3425bp. The TERT PCR product and the pCDH-CMV-MCS-EF1-puro plasmid were digested with XbaI and EcoRI, purified with agarose electrophoresis and ligated by T4 DNA ligase. The ligated product (10μl) was transformed into DH5α competent cells, which were then spread onto an LB plate containing ampicillin (Amp), and incubated at 37°C overnight. Single colonies were picked for colony PCR to identify the right clones by culturing overnight in LB medium followed by plasmid isolation with a plasmid mini-prep reagent kit (Axygen, Hangzhou, China), and digested with XbaI and EcoRI and electrophoresed. For confirmation, the clones that tested positive were further sequenced. The TERT lentiviral expression plasmid pCDH-CMV-MCS-EF1-TERT-puro with the correct sequences was ready for transfection into BMSCs.

### Generation of TERT-BMSCs

SU3-RFP BMSCs were harvested from the mouse bone marrow and confirmed by immunofluorescence staining. They were cultured *in vitro* as described previously. When the cells reached logarithmic growth phase, they were trypsinized and inoculated into 24-well plates at 5×10^4^ cells/well. After 2 days when the BMSCs reached logarithmic growth phase, the cells were infected with lentivirus carrying the expression plasmid pCDH-CMV-MCS-EF1-TERT-puro at a multiplicity of infection (MOI) of 1:8. After 3 days, 5ug/ml puromycin (Solarbio, Beijing, China) was added treated for one week for selection. The positively selected cells were mono-cloned by micropipetting into a 96-well plate and incubated for 7 days to generate single cell cultures clones. Single cell clones with high proliferation capacity, designated as TERT-BMSCs were randomly cultured further.

### Reverse transcription-polymerase chain reaction (RT-PCR)

Total RNA was prepared by Trizol (R0016, Beyotime, Shanghai, China) from 4 cell lines, namely, RAW 264.7 cells (as positive control), TERT-BMSCs, tBMSCs, and BMSCs (approximately 3-5×10^6^ cells). Then, 1μg total RNA was transcribed into cDNA using reverse transcription kit (A3800, Promega, Beijing, China). Then, the cDNA was PCR amplified using above-mentioned TERT-specific primers: 5’-GTC CGA GGT GTC CCT GAG TA-3’, and 5’-GGT TGA AGG TGA GAC TGG CT-3’ according to the following program: 95°C for 30s, 56°C for 30s, and 72°C for 30s for 30 cycles. The PCR products were subjected to 1.5% agarose gel electrophoresis at 100V for 30min and analyzed by gel documentation.

### Western blotting

Total protein from RAW 264.7 cells, TRET-BMSCs, tBMSCs, and BMSCs lysates were prepared by RIPA lysis buffer from RAW 264.7 cells, TRET-BMSCs, tBMSCs, and BMSCs (P0013C, Beyotime, Shanghai, China). Equal amounts of protein lysates (30 μg) were seperated by SDS-PAGE 80 V for 1h at room temperature and then transferred onto a PVDF membrane at100 V for 1h. Then, after washing thrice with PBS, the blots were blocked in 5% non-fat milk at room temperature for 1h and then incubated with rabbit anti-mouse TERT antibody (Abcam, UK) overnight at 4°C. Then, after PBS washing, the blots were incubated with HRP-conjugated anti-rabbit secondary antibody for 30 mins at room temperature. The blots were developed with ECL chemiluminescent system and the images were captured on a Geldoc system.

### Flow cytometry

Single cell suspensions (1×10^6^ cells/ml) of TERT-BMSCs, tBMSCs, and BMSCs were prepared in complete DMEM culture medium containing 10% FBS. The cells (300μl) were stained with 5μl of fluorescent conjugated primary antibodies (CD105, 1:200, Clone number: 8A1; CD90, 1:200, Clone number: IBL-6/23; CD44, 1:150, Clone number: T2-F4; CD29, 1:150, Clone number: KM16; CD45, 1:200, Clone number: IBL-3/16; CD34, 1:200, Clone number: ICO-115; CD-11b, 1:200, Clone number: EPR1344, and CD31, 1:200, Clone number: JC/70A, all from Abcam, UK) for 30min at 37°C in the dark. Then, the samples were washed twice with 1ml of PBS buffer and centrifuged at 1000rpm for 5min. The cells were resuspended in 500μl of PBS buffer and detected by a flow cytometer (BD FACSArial.), and analyzed using CellQuest Pro software. We set isotype control as the appropriate control for the median fluorescence intensity by flow cytometry (Supplementary Figure 1 and Supplementary Table 1).

### Trans-differentiation of BMSCs into osteoblasts and adipocytes

To determine the efficiency of differentiation into osteoblasts, 2×10^4^ tBMSCs, TERT-BMSCs and BMSCs were seeded in 6-well-plate coated with 0.1% gelatin in regular culture medium. After 3 days, the ordinary culture medium was replaced by osteogenic medium (OriCell™, Cyagen), which was changed every three days. After 3 weeks, cells were stained with Alizarin Red S to detect calcium deposition to determine the ability of tBMSCs, TERT-BMSCs and BMSCs to differentiate into osteoblasts. To determine the potential of tBMSCs, TERT-BMSCs and BMSCs to differentiate into adipocytes, 2×10^4^ / cm^2^ cells were grown to confluence in 6-well-plates. Then, they were differentiated in 2 ml adipogenic medium A (OriCellTM, Cyagen) for three days followed by 2 ml adipogenic medium B (OriCellTM, Cyagen) for 1 day. This protocol was repeated 3-5 times (12-20 days) followed by growth in medium B for another 4-7 days until fat droplets became large enough. Then, the cells were stained with Oil Red O and the adipocyte cells were observed. Oil Red O staining was quantified using ImageJ to assess lipid droplet size as previously described [34].

### BMSC growth curve

Single cell suspensions (1×10^5^) of TERT-BMSCs, tBMSCs, and BMSCs were cultured in 25cm^2^ plastic culture flasks in triplicate. When cell density reached 80-90%, the cells were harvested and the mean value was calculated. The population doublings (PDs) at each generation were calculated formulas follows: PDs=log (number of obtained cells/number of inoculated cells)/log_2_ 30. The values were plotted with the culture time as the X-axis coordinate and PD value as longitudinally-axis coordinate.

### BMSC colony formation assay

Single cell suspensions (300 cells/ well) of TERT-BMSCs, tBMSCs, and BMSCs were inoculated into 6-well plates in triplicate. After overnight culture, the numbers of adherent cells were counted. After culturing for 12-14d, the cells were fixed in methanol for 20min and stained with crystal violet for 30min. The cells were washed with tap water, dried and number of colonies was determined under a light microscope (≥50 cells were defined as one cell colony). The colony formation rate was calculated as number of colonies/number of inoculated cells×100%.

### Transwell matrigel cell invasion assay

According the manufacturer’s instructions, a thin-layer of matrigel (1:5) was spread onto the polycarbonate membrane on top chamber of the transwell (Corning, NY, USA) at 60μl/well (24-well, 8μm pore size). Single cell suspensions (3×10^4^ cells in 120μl) of TERT-BMSCs, tBMSCs, and BMSCs cells were seeded in the top chamber, whereas 600μl of DMEM/HG culture medium with 20% FBS was added to the bottom chamber. Each well was plated in triplicate. The cells were incubated at 37°C for 36h. Then, the top chamber was removed, and the cells in the bottom chamber were fixed in 5% paraformaldehyde for 30min, treated with anhydrous ethanol for 20min, and stained with 0.1% crystal violet for 15min. The total numbers of invading cells were counted under light microscope.

### Tumorigenicity assay

TERT-BMSCs, BMSCs, and tBMSCs (1×10^7^ cells) were injected under the right axillary skin of Balb/c athymic nude mice (10 mice for each cell type) and the development of subcutaneous tumors was observed periodically. Tumor tissues were harvested and examined by H&E staining as well as immunofluorescence microscopy.

### Statistical analysis

Data were processed using SPSS software and presented as the mean± standard deviation ($\bar{X}\pm s$). One way ANOVA was performed and P<0.05 was considered statistically significant.

## SUPPLEMENTARY MATERIALS FIGURE AND TABLE


