# Supplementary material for: Human glioma stem-like cells induce malignant transformation of bone marrow mesenchymal stem cells by activating TERT expression
Source: Oncotarget. 2017 Nov 6;8(61):104418–29. doi: 10.18632/oncotarget.22301 (PMC5732816; doi:10.18632/oncotarget.22301)
Supplement: Supplementary file 1 [file oncotarget-08-104418-s001.pdf]

Human glioma stem-like cells induce malignant transformation of bone marrow mesenchymal stem cells by activating TERT expression

SUPPLEMENTARY MATERIALS

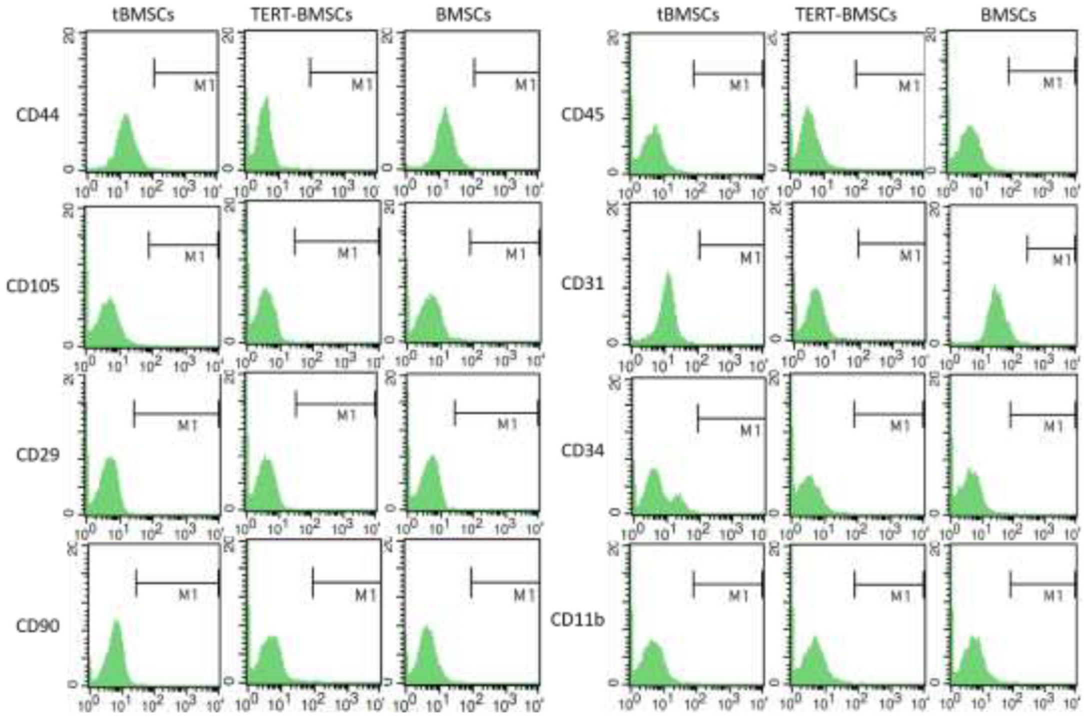

Supplemental Figure 1: Isotype control of cell markers.

Supplemental Table 1: The proportion of expression of the isotype control antibody.

Isotype Control	tBMSCs		TERT-BMSCs		BMSCs	
	ALL%	M1%	ALL%	M1%	ALL%	M1%
CD44 IgG2	90.93	0.03	90.16	0.00	90.08	0.00
CD105 IgG2b	92.95	0.02	97.68	0.01	91.78	0.01
CD29 IgG2a	98.89	0.00	98.43	0.01	98.92	0.00
CD90 IgG1	99.06	0.05	96.97	0.04	90.45	0.00
CD45 IgG1	90.33	0.02	98.09	0.00	92.12	0.02
CD31 IgG1	94.86	0.00	85.79	0.00	92.21	0.01
CD34 IgG1	89.78	0.00	91.37	0.00	90.83	0.03
CD11b IgG	92.59	0.02	92.16	0.02	91.68	0.07
